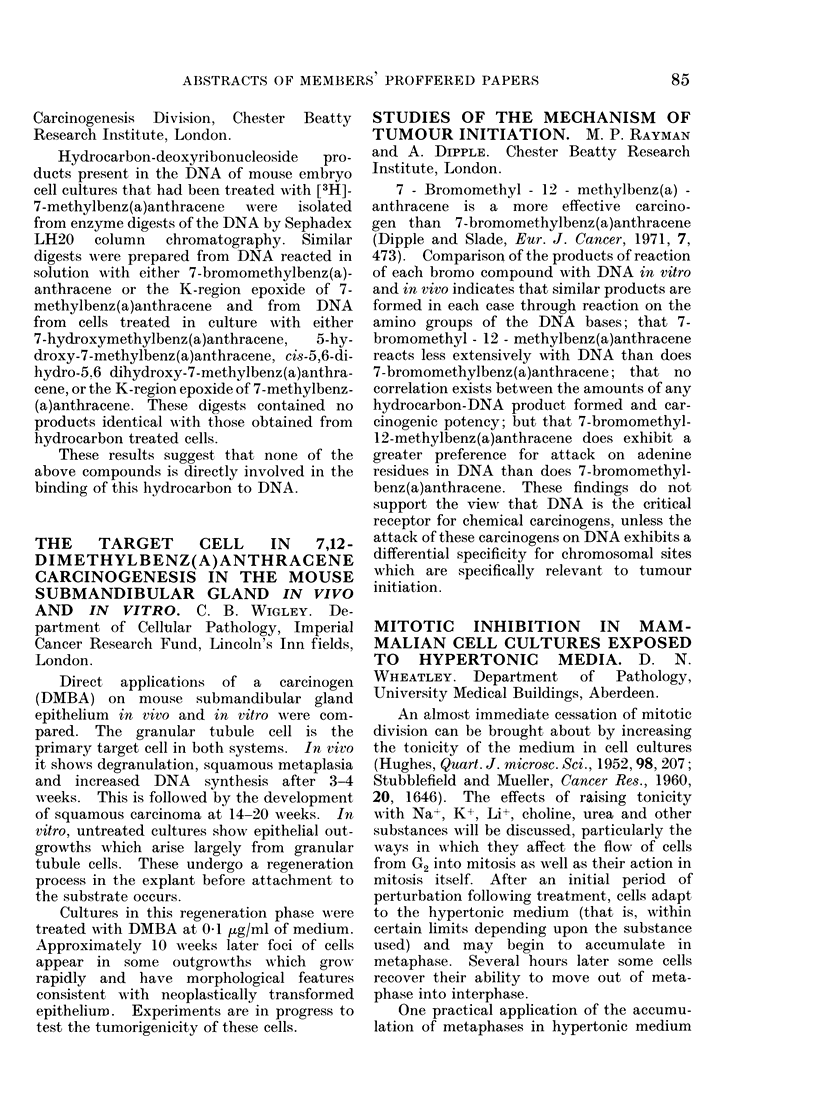# The target cell in 7,12-dimethylbenz(A)anthracene carcinogenesis in the mouse submandibular gland in vivo and in vitro.

**DOI:** 10.1038/bjc.1973.104

**Published:** 1973-07

**Authors:** C. B. Wigley


**THE TARGET CELL IN 7,12-DIMETHYLBENZ(A)ANTHRACENE CARCINOGENESIS IN THE MOUSE SUBMANDIBULAR GLAND *IN VIVO* AND *IN VITRO*.** C. B. Wigley. Department of Cellular Pathology, Imperial Cancer Research Fund, Lincoln's Inn fields, London.

Direct applications of a carcinogen (DMBA) on mouse submandibular gland epithelium *in vivo* and *in vitro* were compared. The granular tubule cell is the primary target cell in both systems. *In vivo* it shows degranulation, squamous metaplasia and increased DNA synthesis after 3–4 weeks. This is followed by the development of squamous carcinoma at 14–20 weeks. *In vitro*, untreated cultures show epithelial outgrowths which arise largely from granular tubule cells. These undergo a regeneration process in the explant before attachment to the substrate occurs.

Cultures in this regeneration phase were treated with DMBA at 0·1 $\mu$g/ml of medium. Approximately 10 weeks later foci of cells appear in some outgrowths which grow rapidly and have morphological features consistent with neoplastically transformed epithelium. Experiments are in progress to test the tumorigenicity of these cells.